# Magnetostrictive Properties of Mn_0.70_Zn_0.24_Fe_2.06_O_4_ Ferrite

**DOI:** 10.3390/ma11101894

**Published:** 2018-10-03

**Authors:** Adam Bieńkowski, Roman Szewczyk

**Affiliations:** Institute of Metrology and Biomedical Engineering, Warsaw University of Technology, 02-525 Warsaw, Poland; a.bienkowski@mchtr.pw.edu.pl

**Keywords:** power ferrites, Mn-Zn ferrites, magnetostriction

## Abstract

This paper presents the results of measurements of magnetostrictive properties of Mn_0.70_Zn_0.24_Fe_2.06_O_4_ ferrite for power applications. Frame-shaped samples were used for measurements to guarantee a uniform magnetizing field and magnetostrictive strain distribution. Magnetostrictive hysteresis loops were measured by semiconductor strain gauges. The results indicate that the magnetostrictive characteristic of Mn_0.70_Zn_0.24_Fe_2.06_O_4_ ferrite is non-monotonic and magnetostriction changes have opposite signs for higher values of the magnetizing field.

## 1. Introduction

Magnetostriction is the most important magnetomechanical effect connected with the changes of the size of sample made of magnetic material subjected to a magnetizing field. In spite of the fact that the magnetostriction effect was described first in 1847 by Joule [1], this phenomenon is still not fully understood. Previously presented models of magnetostriction [2,3] are rather a general, qualitative explanation of magnetostriction mechanisms. Quantitative understanding of the magnetostrictive characteristics requires quantum effects-based models [4], which are still under development.

In spite of the lack of sufficient quantitative models, magnetostriction has a great technical importance. Magnetostrictive strain is the main source of acoustic noise generated by transformers [5]. Moreover, magnetostriction can cause noise in signals from inductive components. However, magnetostriction may be utilized in the development of high power acoustic generators [6] (mostly ultrasonic) as well as acoustic waves, enabling a technological breakthrough in the development of two-phase fluids level sensors [7].

Manganese–Zinc ferrites (Mn–Zn ferrites) are commonly used as cores for electronic transformers, as well as cores of inductive components for switching mode power supplies. For this reason, the magnetostrictive properties of such ferrites are important, due to possible acoustic noise generation from transformers as well as additional electronic noise in switching mode power supplies’ output signal. In spite of this fact, the magnetostrictive characteristics of such ferrites were still not presented. This lack in the state of the art is connected with difficulties in the measurement of the magnetostrictive characteristics of such ferrites connected with relatively small values of magnetostriction, as well as technical problems with the measurement of magnetostriction of commonly used ring-shaped samples.

This paper is filling the gap in the state of the art connected with the magnetostrictive characteristics of Mn–Zn ferrites for power application. The magnetostrictive characteristics of Mn_0.70_Zn_0.24_Fe_2.06_O_4_ ferrite are presented together with a quantitative explanation of the mechanisms behind the non-monotonic magnetostrictive characteristics of this material.

## 2. Materials and Methods

### 2.1. Frame-Shaped Sample

Among the different methods of measurements of magnetostriction [8], the strain gauge method seems to be the most accurate and reliable method of measurement of this phenomenon in bulk magnetic materials. However, the measurement of magnetostrictive strain of ring-shaped samples by strain gauges is difficult. In this case, strain gauge is subjected to both elongation and bending. To avoid this problem and provide a uniform distribution of both the magnetizing field as well as magnetostrictive strain, isotropic frame-shaped samples [9] made of Mn_0.70_Zn_0.24_Fe_2.06_O_4_ ferrite for power applications were produced on demand by POLFER Company in specialized forms for sintering.

**Figure 1:**
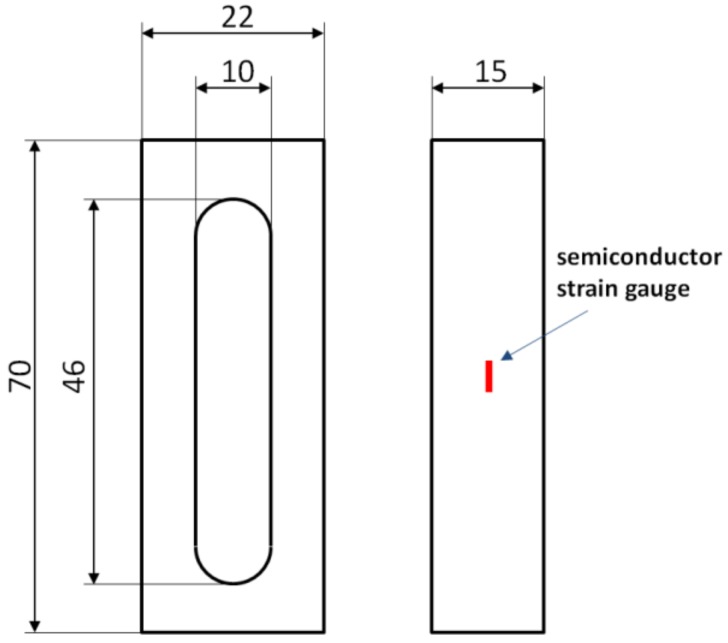
Frame-shaped sample made of Mn_0.70_Zn0._24_Fe_2.06_O_4_ ferrite for power applications.

A general view of the frame-shaped samples made of Mn_0.70_Zn_0.24_Fe_2.06_O_4_ ferrite, used for investigation, is presented in Figure 1. The samples had 70 mm length, 22 mm width, and 15 mm thickness. Strain gauge was attached in the middle of the column, whereas the second column was wound by the magnetizing and sensing windings with 15 and 50 turns, respectively.

### 2.2. Method of Measurements

Magnetostrictive strain was measured by semiconductor strain gauges AP120-10-12 (VTS, Zlin, Slovak Republic), in conjunction with an MT-12 strain gauge transducer (MERATRONIC, Warsaw, Poland). Semiconductor strain gauges exhibit sensitivity which is about 60 times higher than that of standard metal foil strain gauges. However, sensitivity of strain gauges may be nonlinear in a wide range of measurements. For this reason, the influence of the shrinkage of glue on sensitivity should be considered according to the recommendations of producer [10].

**Figure 2:**
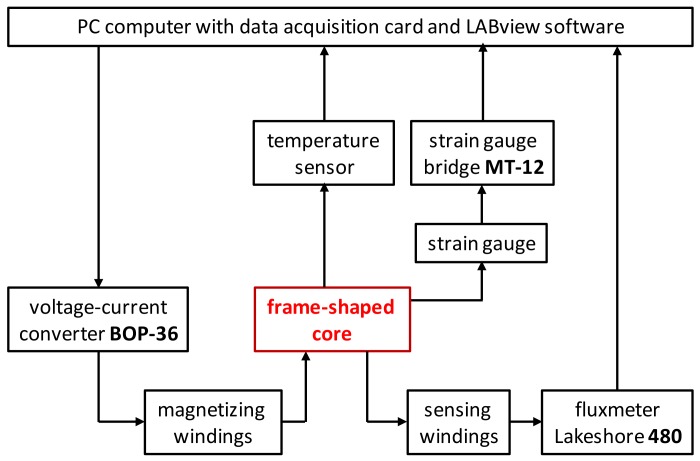
Schematic diagram of the developed system for measurements of both magnetostrictive and magnetic hysteresis loops.

The measurements were carried out on the specially designed PC-controlled system at room temperature. Figure 2 presents a schematic diagram of this system. The system was controlled by a PC equipped with an NI PCI-6221 DAQ card. Specialized software was developed in a LabView environment, enabling synchronized sample magnetization as well as measurements of both flux density and magnetostrictive strain.

The voltage driving signal from the DAQ card was converted to the current in a KEPCO BOP36-6M voltage-current converter (KEPCO, Flushing, NY, USA). The measuring setup enables simultaneous measurements of flux density *B* and magnetostrictive strain *λ*. For this reason, the sensing windings of the sample were connected to a Lakeshore 480 fluxmeter and the semiconductor strain gauges were connected to a specialized MT-12 bridge, enabling offset compensation as well as sensitivity adjustments. During the measurements, the temperature of the sample was also monitored by a thermocouple.

## 3. Results

**Figure 3:**
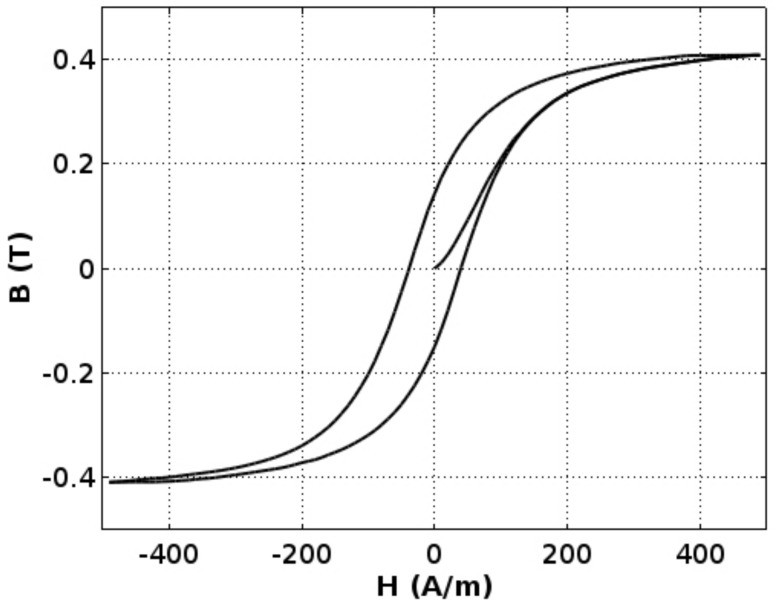
Magnetic hystresis loop *B(H*) of the Mn_0.70_Zn_0.24_Fe_2.06_O_4_ ferrite for power applications.

Magnetic hysteresis loop *B(H)* of the Mn_0.70_Zn_0.24_Fe_2.06_O_4_ ferrite for power applications is presented in the Figure 3. The saturation flux density *B_s_* of this ferrite is about 0.45 T, which is in line with typical values for such materials. Moreover, the coercive field *H_c_* is about 25 A/m, which confirms that frame-shaped samples exhibit similar properties to typical ring-shaped samples [11].

**Figure 4:**
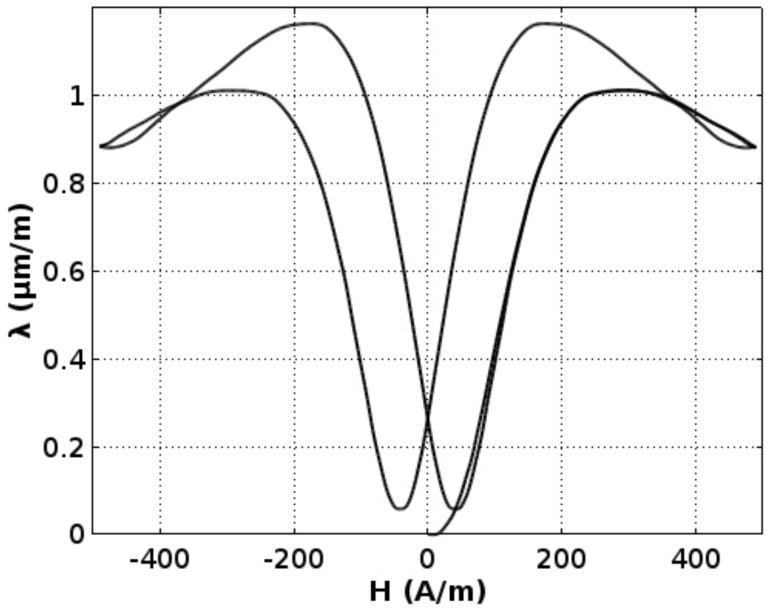
Magnetostrictive hystresis loop *λ(H)* of the Mn_0.70_Zn_0.24_Fe_2.06_O_4_ ferrite for power applications.

**Figure 5:**
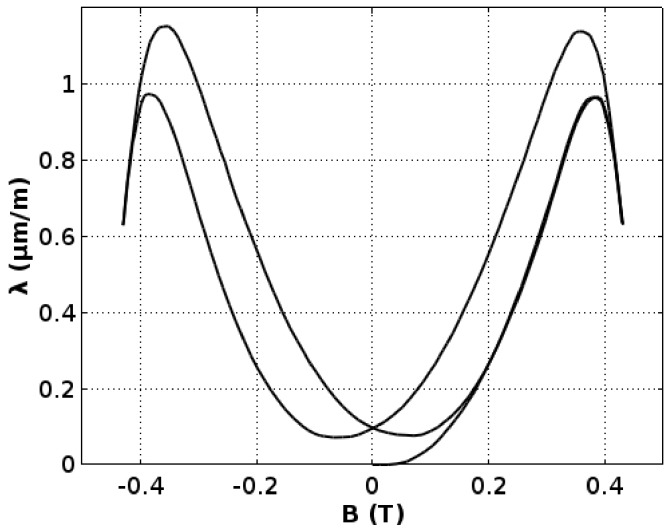
Magnetostrictive hystresis loop *λ(B)* of the Mn_0.70_Zn_0.24_Fe_2.06_O_4_ ferrite for power applications.

Figure 4 presents magnetostrictive *λ(H)* hysteresis loop, whereas Figure 5 presents *λ*(B) dependence. In both characteristics, the “lift-off” phenomenon [2] may be observed. This phenomenon is connected with the fact that, after magnetization from a demagnetized state, the absolute value of magnetostrictive strain never comes back to zero.

The maximal value of magnetostrictive strain *λ*_max_ of the Mn_0.70_Zn_0.24_Fe_2.06_O_4_ ferrite for power applications is slightly below 1.2 μm/m. However, the saturation magnetostriction *λ*_s_ is about 0.6 μm/m. Moreover, for the flux density *B*, about 0.35 T (and magnetizing field *H* about 200 A/m) magnetostriction changes switch its sign. As a result, maximal magnetostriction is significantly lower than magnetostriction in saturation. This phenomenon may be misleading in the case of measuring methods focused on saturation magnetostriction measurements.

## 4. Qualitative Explanation of Results

**Figure 6:**
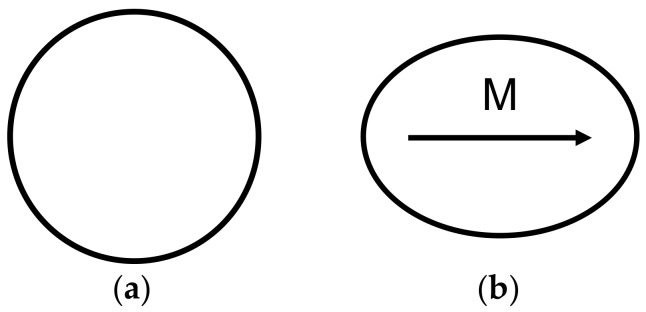
Crystalized sphere made of paramagnetic material ((**a**) over Curie temperature) re-shapes into ellipsoid made of magnetic material ((**b**) below Curie temperature).

**Figure 7:**
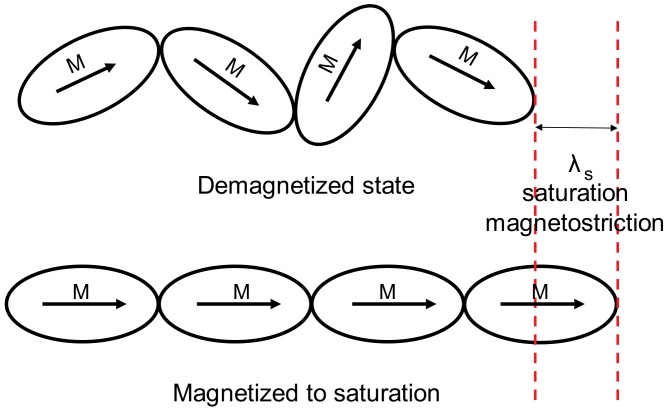
Simplified qualitative explanation of saturation magnetostriction *λ_s_* (for *λ_s_* > 0).

The quantitative explanation of the magnetostriction phenomenon is sophisticated and not fully understood [12]. However, the simple qualitative model of magnetostriction is presented in Figure 6 and Figure 7.

Due to the fact that Curie temperature is significantly lower than the temperature of crystallization, magnetic material crystallizes in a paramagnetic state. During cooling after crystallization, the material starts to exhibit ferromagnetic properties with a domain structure for temperatures below Curie temperature. Transformation from a paramagnetic to a ferromagnetic state influences the total free energy of magnetic material, causing deformation. This deformation is known as spontaneous magnetostriction [12]. As a result, the sphere changes into an ellipsoid, as presented in Figure 6. The simplest explanation for the magnetostrictive phenomenon is the rotation of these ellipsoids (as it is presented in Figure 7 for materials with positive saturation magnetostriction *λ_s_*) during the magnetization of the material, from a demagnetized state to saturation.

Quantitative analysis [2] of this model leads to the conclusion that magnetostriction *λ(B)* dependence is given by the square dependence:

(1) $$\lambda \left(B\right)=aB^{2}$$

where *a* is constant. Such a model is useful for technical applications [13]; however, it doesn’t explain the “lift-off” phenomenon as well as the hysteresis on the *λ(B)* characteristics. However, in many cases, it represents the *λ(H)* hysteresis loops quite well.

**Figure 8:**
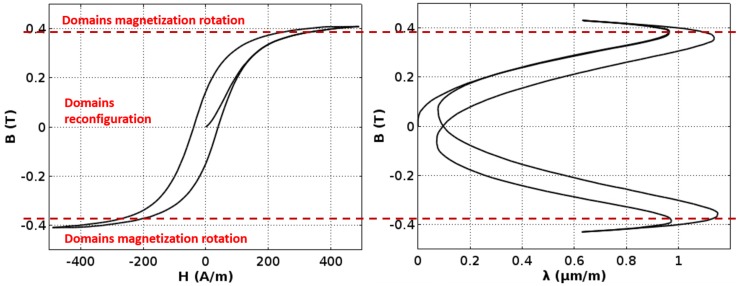
Simplified qualitative explanation of saturation magnetostriction *λ_s_.*

It should be stressed that the simplified model given by Equation (1) is not suitable for an explanation of the phenomenon of the switch of the sign of magnetostrictive changes, as it is presented in Figure 4 and Figure 5. A simplified, qualitative explanation of this effect is given in the Figure 8. Equation (1) is valid only for a magnetization mechanism connected with changes of domains configuration. This mechanism is dominating the magnetization process in the range of magnetizing field *H* from −200 A/m up to 200 A/m. For a higher absolute value of the magnetizing field, the magnetization mechanism is connected with a magnetization rotation from the easy to hard axis in domains connected with single crystals. This mechanism is connected with a different type of magnetostriction strain (what was observed in single crystals [14]) and can exhibit a linear dependence with a different sign than magnetostriction due to changes in domains configuration.

In addition, switching the sign of the magnetostrictive changes may be the reason behind the appearance of fourth harmonics in acoustic noise generated by power transformers with cores made of Mn–Zn ferrites. For this reason, magnetostrictive characteristics of such ferrites should be carefully investigated.

## 5. Conclusions

The presented results confirm that semiconductor strain gauges enable measurements of the magnetostrictive characteristics of Mn–Zn ferrites for power applications. The results of these measurements indicate that the magnetostrictive characteristics of the Mn_0.70_Zn_0.24_Fe_2.06_O_4_ ferrite for power applications are switching the sign of the magnetostrictive changes for flux density of about 0.35 T. This phenomenon is connected with the change of magnetization mechanism from domain walls movement to magnetization rotation, from the easy to hard axis of single crystals.

## References

[1] Joule J.P. (1847). On the Effects of Magnetism upon the Dimensions of Iron and Steel Bars. Lond. Edinb. Dublin Philos. Mag. J. Sci..

[2] Sablik M., Jiles D.C. (1993). Coupled magnetoelastic theory of magnetic and magnetostrictive hysteresis. IEEE Trans. Magn..

[3] Wijn H.P.J., Gorter E.W., Esveldt C.J., Geldermans P. (1954). Conditions for square hysteresis loops in ferrites. Philips Tech. Rev..

[4] Odkhuu D., Taivansaikhan P., Yun W., Won S., Hong S. (2014). A first-principles study of magnetostrictions of Fe_3_O_4_ and CoFe_2_O_4_. J. Appl. Phys..

[5] Moses A.J., Anderson P., Phophongviwat T., Tabrizi S. Contribution of magnetostriction to transformer noise. Proceedings of the 45th International Universities Power Engineering Conference UPEC2010.

[6] Kaczkowski Z., Kisdi-Koszo E., Potocky L. (1994). Ultrasound Velocities in Fe_81.5_Cr_4.5_B_14_ Metallic Glasses Produced in Magnetic Field. IEEE Trans. Magn..

[7] Li Y.B., Sun L.Y., Jin S.J., Sun L.B. Development of Magnetostriction Sensor for on-line Liquid Level and Density Measurement. Proceedings of the 6th World Congress on Intelligent Control and Automation.

[8] Squire P.T. (1994). Magnetomechanical measurements of magnetically soft amorphous materials. Meas. Sci. Technol..

[9] Bieńkowski A. (1992). Some problems of measurements of magnetostriction in ferrites under stresses. J. Magn. Magn. Mater..

[10] Strain gauges. https://vtsz.cz/en/strain-gauges.

[11] Bieńkowski A., Rożniatowski K., Szewczyk R. (2003). Effects of stress and its dependence on microstructure in Mn-Zn ferrite for power applications. J. Magn. Magn. Mater..

[12] Jiles D.C. (1998). Introduction to Magnetism and Magnetic Materials. Chapman Hall Lond..

[13] Calkins F.T., Smith R.C., Flatau A.B. (2000). Energy-Based Hysteresis Model for Magnetostrictive Transducers. IEEE Trans. Magn..

[14] Grössinger R., Turtelli R.S., Mehmood N. (2014). Materials with high magnetostriction. Mater. Sci. Eng..

